# Evaluation of role of hyperbilirubinemia as a new diagnostic marker of complicated appendicitis

**DOI:** 10.1186/s12876-021-01614-x

**Published:** 2021-01-28

**Authors:** Sabyasachi Bakshi, Nilay Mandal

**Affiliations:** 1 Department of General Surgery, Bankura Sammilani Medical College and Hospital, Bankura, West Bengal 722102 India; 2 Kathghara Lane, Sonatuli, PO, Hooghly, West Bengal 712103 India

**Keywords:** Total serum bilirubin, Hyperbilirubinemia, Acute appendicitis, Gangrenous/perforated appendicitis, Ultrasonography, Appendix diameter, Fecolith, Complicated appendicitis

## Abstract

**Background:**

In appendicitis, elevated intra-luminal pressure and ischemic necrosis of mucosa causes tissue gangrene or perforation. This leads to cytotoxin facilitated progressive bacterial invasion or translocation into the hepatic parenchyma through portal system. This phenomenon interferes with the bilirubin excretion into the bile canaliculi. In the present study, establishment of a possible role of hyperbilirubinemia as a marker of gangrenous/perforated appendicitis has been studied.

**Methods:**

After matching the inclusion and exclusion criteria, all cases of clinically diagnosed acute appendicitis were taken for this prospective, single center, observational study. Per-operative diagnosis was confirmed by histopathological examination.

**Results:**

Out of 110 subjects of acute appendicitis 41 subjects (37.27%) had hyperbilirubinemia. Out of 35 subjects diagnosed as complicated appendicitis 32 subjects (91.42%) had raised total bilirubin levels, while the remaining 03 (8.58%) had normal levels. Among 75 subjects diagnosed as acute simple appendicitis 09 subjects (12%) had raised total bilirubin level, while the remaining 66 subjects (88%) had normal levels. It was Mixed Type of Hyperbilirubinemia in gangrenous/perforated appendicitis. The sensitivity of Total serum bilirubin in predicting complicated appendicitis was found 91.43% (76.942% to 98.196%), where as the specificity of this test was 88.00% (78.439% to 94.363%). positive predictive value and negative predictive value were 78.03% and 95.65% respectively. Positive likelihood ratio and negative likelihood ratio were found to be 7.619 and 0.097 respectively taking prevalence of complicated appendicitis be 31.80%. Receiver Operating Characteristic curve was obtained which shows optimal criterion at Total Bilirubin Level 1.06 mg/dl where sensitivity was 91.43% and specificity was 97.33% at 95% confidence interval with 31.8% disease prevalence.

**Conclusions:**

This is to conclude that Serum bilirubin level estimation, which is a simple, cheap and easily available laboratory test, can be added to the routine investigations in clinically suspected cases of acute appendicitis for early diagnosis of complications.

*Trial registration* Registered with Clinical Trials Registry-India (ICMR-NIMS) with Registration number CTRI/2019/05/018879 Dated 01/05/2019. This was a prospective trial. Trial URL: http://ctri.nic.in/Clinicaltrials/pdf_generate.php?trialid=33113&EncHid=99780.32960&modid=1&compid=19%27,%2733113det%27.

## Background

Hyperbilirubinemia has also been found during some infective diseases involving organs other than liver [1]. Neonates are more susceptible to develop hyperbilirubinemia following gram-negative bacterial infections. Severe intra-abdominal infection in adults has also been associated with development of hyperbilirubinemia [2]. The vermiform appendix is important in surgical practice mostly due to its propensity for inflammation resulting in acute appendicitis. Worldwide acute appendicitis is the most common surgical emergency, affecting the abdomen. An emergency appendectomy is the most frequently performed abdominal operation and often it is the first major operative procedure done by a surgeon [3]. Overall lifetime risk of developing appendicitis is approximately 7% (8.6% for males and 6.7% for females) [4, 5]. The male: female ratio is 1.4:1 (range is M:F = 1:1 to 3:1) [5]. The incidence of acute appendicitis is decreasing steadily since late 1940 with present incidence rate approximately up to 110 (starting from 55.3 in females and 68.8 in males) cases per 10,0000 population per year [6].

Appendicitis commonly occurs in young adults (the highest incidence, approximately 40%, in 2nd decade of life i.e. 10–19 years and 70% of the subjects are less than 30 years old.) [7, 8]. Acute appendicitis is relatively rare at the extreme of age [9, 10]. Most subjects with acute appendicitis present with classic signs and symptoms for the ease of diagnosis. But in some atypical presentations diagnostic confusion and delay in treatment may occur. Crohn's disease, ectopic pregnancy, diverticulitis, endometriosis, mittleschmerz, mesenteric adenitis, omental torsion, pelvic inflammatory diseases, ruptured ovarian cyst, urinary tract infection may mimic acute appendicitis. Worldwide mean value for difference in diagnostic error rate, ranges from 12 to 23% and 24–42% respectively in men and women. Error occurs mostly in whites (74%), while it is lesser in darker complexions (5%) [9].

Surgical delay in a prompt management of the subjects with appendicitis (not with perforation, in particular), either due to delay in presentation (particularly in males with retrocaecal or retroileal position) or misjudgment, leads to dread complications like gangrenous changes and perforation of the appendix. Gangrene or perforation further leads to more complications like appendicular abscess formations, localized/generalized peritonitis, fecal fistula formation, intestinal obstruction due to adhesion formation, portal pyemia, sepsis and sterility in women of child-bearing age (though recent studies denies it as a major risk factor) with overall increased morbidity and prolonged hospital stay [5, 11]. In adults, the incidence of appendicular perforation is 13–37% [12]. The risk is higher in extreme of ages (45% in under 5 years age group and 51% in over 65 years age group) [7, 10]. The mortality rate for uncomplicated, non-perforated appendicitis is 0.1–0.5% while that of perforated appendicitis is much higher, ranging from 3% overall to as high as 15% in elderly subjects [7]. On the contrary, in case of diagnostic difficulties and atypical presentations if appendectomy is performed based on clinical suspicion only, may increase the number of unnecessary appendectomies (up to 20%) [13]. The rate of negative appendectomy (mostly due to pelvic inflammatory conditions) is 35 to 45% in women of child bearing age [14]. Unnecessary appendectomy caries a small risk of wound sepsis and the subsequent adhesive intestinal obstruction and occurrence of incisional hernia.

In spite of numerous advances in the diagnosis, evolved in the last 125 years, still now acute appendicitis continues to be a diagnostic challenge for surgeons and it remains mostly a clinical Diagnosis. Additional laboratory tests, scoring systems, Ultrasonography (sensitivity of 0.86 and specificity of 0.81 in experienced hand), Multi Detector computed tomography (MDCT, with sensitivity and specificity of 0.94 and 0.95 respectively), scintigraphy, Magnetic Resonance Imaging (specially in pregnancy) and diagnostic laparoscopy has been used which may support the primary clinical assessment to reach the diagnosis [15–18].

Several diagnostic scoring systems such as the Alvarado score (Scale 0–10), modified Alvarado score, Pediatric Appendicitis Score (PAS; scale 0–10), Rajalsteri Pengiran Anak Saleha Appendicitis (RIPASA) score for use in Asian patients (Scale 0–14), and Appendicitis Inflammatory Response Score (AIRS; scale 0–12) are commonly used in clinically suspected cases [16, 19–22]. But these scoring systems do not assess the risk of complications like appendicular gangrene or perforation. None of the above mentioned scores use hyperbilirubinemia as a marker. Some studies had showed that risk of appendicular perforation increases three times in subjects with total serum bilirubin levels more than 1 mg/dl [9].

Obstruction of appendix lumen, forming a closed loop, either by fecolith or mucosal edema is the major cause of pathological changes in acute appendicitis. Fecolith increases the severity of appendicitis, like 40% of uncomplicated, 65% of Gangrenous Appendicitis and 90% of Perforative Appendicitis has been found to be associated with presence of fecolith [23, 24]. Appendix perforation occurs mostly distal to the point of luminal obstruction along the anti-mesenteric border. The appendix perforates about 12 to 48 h after the onset of acute appendicitis and is accompanied by an abscess cavity walled off by the small intestine and the omentum. Rarely free pertoration of the appendix into the peritoneal cavity occurs, which may be accompanied by peritonitis and septic shock and may be complicated by subsequent formation of multiple intraperitoneal abscesses.

**Fig. 1:**
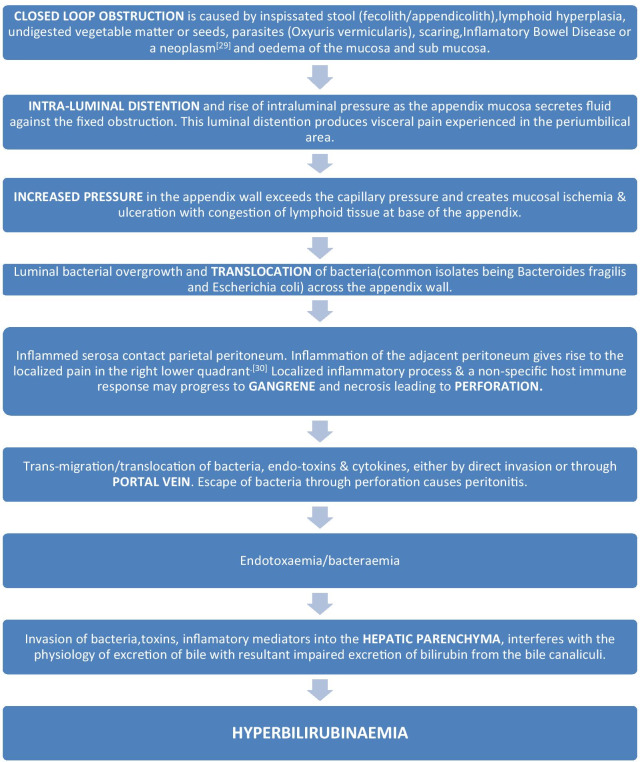
Sequence of events in acute appendicitis giving rise to Hyperbilirubinemia

Wangensteen, postulated that mucosal folds and a sphincter like orientation of the muscle Fibres at the appendiceal orifice makes the appendix susceptible to obstruction. He proposed sequence of events to explain appendicitis as depicted in Fig. 1 [9]. The flora of the inflamed appendix contains more bacterial load (anaerobes in 60% cases, peptostreptococcus, pseudomonas, bacteroides splanchnicus/intermedius, lactobacillus) than usual species in normal appendicitis [25]. Fusobacterium necrophorum /nucleatum, which is absent in normal situation, have been identified in 62% of inflamed appendices [26]. Bacteroides fragilis and Escherichia coli are the most frequently isolated bacteria in appendicitis [27]. Nearly 80% of blood supply to the liver comes from portal venous system, which carries absorbed substances including bacteria and toxins from intestine. Normally, small amount of bacteria and their toxic products get cleared after entering liver by detoxification and immunological action of reticulo-endothelial system. But kupffer cells may fail to clear the over abundant bacterial load in case of complicated acute appendicitis which in turn damage hepatocytes and rise serum bilirubin level. When there is bacteraemia, it leads to endotoxemia with resultant impaired excretion of bilirubin from the bile canaliculi [27]. Cytokines e.g. interleukin-6 (IL6), tumor necrosis factors (TNF) are also been considered to depress excretory functions of the liver resulting in hyperbilirubinemia without rise in liver enzymes level [28].

Aim of the present study is to evaluate the diagnostic role and predictive value of elevated total serum bilirubin level as a diagnostic parameter of complicated (gangrenous or perforated) appendicitis. Whether direct bilirubin, indirect bilirubin or both is increased in case of complicated appendicitis and their relation with complicated appendicitis were also studied. The relationship of other patient related parameters with complicated appendicitis were also evaluated in the present study.

## Methods

This is an institution based (single center) prospective, observational study. The study population comprised of clinically suspected (110) subjects, satisfying the inclusion and exclusion criteria mentioned below. The primary data for this study were the investigation reports of the subjects.

### Inclusion criteria for the study group

All cases of clinically diagnosed acute appendicitis of age 5 years and above, scheduled for appendectomy at emergency surgical unit of this hospital, were taken for this study.

### Exclusion criteria for the study group

A. Subjects with age below 5 years.
B. Subjects with appendicular lump formation.
C. All subjects documented to have a past history of
  1. Chronic liver disease with hyperbilirubinemia.
  2. Chronic alcoholism (intake of alcohol of > 40 g/day for Men and > 20 g/day in Women for 10 years)
  3. Hemolytic disease or Gilbert’s Syndrome (conjugated hyperbilirubinemia with prevalence rate of 6%), Dubin-Johnson syndrome.
  4. Acquired or Congenital Biliary Disease.
D. Subjects with Acute hepatitis (viral/positive HbsAg and unknown).
E. Subjects with history of gastro intestinal malignancy.
F. History of hepatotoxic drug use either past or recent.
G. All subjects with cholelithiasis, benign recurrent intrahepatic cholestasis.

### Determination of reference value of serum bilirubin level in study population

Serum bilirubin levels of healthy people (representing similar population with study participants) who were electively admitted with non gastrointestinal diseases (like benign skin lesions, fibroadenoma breast etc.) were checked. The mean value of their serum bilirubin level (1.00 mg/dl) was taken as normal reference value for the study population.

### Study procedure

All participants in the study were clinically evaluated by detailed history and thorough clinical examination on initial contact. After clinical confirmation of acute appendicitis, the following investigations were done for all participants:

1. Routine blood investigations (i.e. complete blood count, platelet count etc.).
2. Peripheral blood smear to rule out hemolytic anemia.
3. Serum Bilirubin (Total and Direct bilirubin).
4. Liver Enzymes, which include—ALT (Alanine transaminase), AST (Aspartate transaminase), ALP (Alkaline phosphatase).
5. Seropositivity for HbsAg, HIV, HCV.
6. Serum CRP Level.
7. Fasting blood sugar, renal function test.
8. ECG, digital chest X-ray—PA view and USG Scan of whole abdomen (specially to assess the appendix diameter, peri appendicular collection and presence of fecolith).

Blood samples were drawn within half an hour of presentation in the hospital and radiological investigations were done within 2 h of admission. All the available data were recorded. After initial stabilization, these subjects were operated (emergency open appendectomy). Finally, clinical diagnosis was confirmed by post operative histopathological examination. Histopathological examination was considered final in diagnosing and categorizing subjects as.

1. Negative for the study (having normal appendix or acute uncomplicated appendicitis)
2. Positive for the study (Acute Appendicitis with perforation/gangrene).

The post-operative follow up was done for a period of 5 days in hospital and at least one more Out-Patients Department visit on 7th Post operative day. The serum bilirubin level was rechecked on 7th post-operative day, during follow-up in OPD. Their clinical data were compiled and analyzed.

### Accepted standard normal ranges for this study were like the following

- Serum Bilirubin: Total = 0.3–1.0 mg/dl, Direct Bilirubin = 0.1–0.3 mg/dl.
- Liver Enzymes: ALT = 0–40 U/l, AST = 0–40 U/l, ALP = 30–130 U/l.

The statistical analysis was carried out using available standard statistical software (SPSS 23). All statistical tests was one tailed and *p* value < 0.05 was taken as significant.

## Results

Total number of cases was 110, all completed the follow up. Systematic analysis of all collected data revealed the following

A. Baseline characteristics: Incidence of complicated appendicitis among subjects undergoing appendectomy was found as following

(I) Gender specific

Out of total 110 subjects of acute appendicitis, enrolled for the study, 66 subjects (60%) were male while the remaining 44 subjects (40%) were female. So, male:female ratio was 1.5:1. Overall occurrence of complicated (gangrenous/perforated on the basis of intra operative as well as histopathology report) appendicitis was 31.8% (35 out of them 15 were males and 20 females) and 68.2% (75 out of them 51 were males and 24 were females) subjects had appendicitis without complications. Complicated appendicitis was more (57.2%) in females than males (42.8%). But 45.45% of all females had complicated appendicitis while among males the rate of complication was 22.72%. 44% males and 25% females had simple appendicitis while 12% male and 8% females had gangrenous appendicitis. Perforated appendicitis was found in 4% males and 7% females (Table 1).

(II) Age specific

**Table 1:** Showing age and gender specific cumulative distribution of study population having acute appendicitis and its complications

Complicated appendicitis	Gender				Total				Χ^2^, df				*p* value			
	Male N(%)		Female N(%)													
Absent	51(68)		24(32)		75(68.2%)				6.229, 1				0.0126			
Present	15(42.8)		20(57.2)		35(31.8%)											
Total	66(60)		44(40)		110(100%)											

Age group (years)	Total acute appendicitis (N = 110)					Complicated appendicitis (n = 35)					Uncomplicated appendicitis (n = 75)					Overall complication rate in particular age-group (%)
	Male	Female	Total	Total %	Cumulative %	Male	Female	Total	Total %	Cumulative%	Male	Female	Total	Total %	Cumulative %	
< 10	10	6	16	14.54	14.54	4	3	7	20.00	20.00	6	3	9	12	12.00	43.75
10–19	14	17	31	28.18	42.72	9	13	22	62.85	82.85	5	4	9	12	24.00	70.96
20–29	24	9	33	30.00	72.72	0	3	3	8.57	91.42	24	6	30	40	64.00	9.09
30–39	7	6	13	11.82	84.54	1	0	1	2.86	94.28	6	6	12	16	80.00	7.69
40–49	8	2	10	9.11	93.64	0	0	0	0.00	94.28	8	2	10	13.35	93.35	0
50–59	2	2	4	3.59	97.14	1	0	1	2.86	97.14	1	2	3	4	97.35	25
> 60	1	2	3	2.76	100	0	1	1	2.86	100	1	1	2	2.65	100	33
Total	66	44	110	100		15	20	35	100				75	100		

The overall mean age of all 110 subjects was 23.5 ± 13.5 years (range 5–62 years). The mean age for males was 24.34 ± 12.70 years (range 5–60 years) and the mean age for females was 22.29 ± 14.84 years (range 6–62 years). Majority (almost 83%) of the cases belongs to first two decades (5–19 years) of life. But occurrence rate of complicated appendicitis was more at the extremes of ages. The ratio of complicated appendicitis among all study population were 43.75% (in < 10 years age group), 70.96% (in 10–19 years age group), 25% (in 50–59 years age) and 33% (in age group more than 60 years) (Table 1).

B. Relationship of direct bilirubin and indirect bilirubin with complicated appendicitis

Out of 110 subjects of acute appendicitis 41 subjects (37.27%) had hyperbilirubinemia (raised serum total bilirubin level > 1 mg/dl). Out of 35 subjects diagnosed as complicated appendicitis 32 subjects (91.42%) had raised total bilirubin levels (> 1.0 mg/dl). While the remaining 03 subjects (8.58%) had normal levels (< 1.0 mg/dl). Among 75 subjects diagnosed as acute simple appendicitis 09 subjects (12%) had raised total bilirubin level (> 1.0 mg/dl), while the remaining 66 subjects (88%) had normal levels (< 1.0 mg/dl).

The mean Total Bilirubin Level for all subjects with acute uncomplicated appendicitis was 0.79 ± 0.16 mg/dl (range being 0.45–1.20 mg/dl) and that for complicated appendicitis was 1.39 ± 0.26 mg/dl (range being 0.68–2.20 mg/dl) value was statistically significant *p* < 0.05. Hence, it was seen that subjects with complicated (perforated/gangrenous) appendicitis had higher level of total bilirubin as compared to that of uncomplicated acute appendicitis (Table 2).

**Table 2:** Distribution of study population according to total serum bilirubin (n = 110), direct and indirect bilirubin (n = 110) and distribution of pre-operative and post-operative mean bilirubin level in study population

Complication	< 1 mg/dl	> 1 mg/dl	Total	Χ^2^, df	*p* value		
*Serum total bilirubin level*							
Absent	66	9	75	64.39, 1	< 0.05		
Present	3	32	35				
Total	69	41	110				

Type of bilirubin	Type of appendicitis	Number of cases	Mean	SD	t	df	*p* value
Total	Uncomplicated	75	0.79	0.16	14.63	108	0.001
	Complicated	35	1.39	0.26			
Direct	Uncomplicated	75	0.43	0.1	12.73	108	0.016
	Complicated	35	0.72	0.13			
Indirect	Uncomplicated	75	0.36	0.08	13.65	108	0.021
	Complicated	35	0.66	0.14			

Total bilirubin level	(N = 110)		Mean	SD	t	df	*p* value
Pre-operative (mean)	Complicated appendicitis (n = 35)	1.39	0.98	0.346	8.85	109	< 0.05
	Uncomplicated appendicitis (n = 75)	0.69					
Post-operative on Day 7 (mean)	Complicated appendicitis (n = 35)	0.79	0.69	0.104			
	Uncomplicated appendicitis (n = 75)	0.69					

The mean DIRECT BILIRUBIN for uncomplicated appendicitis subjects was 0.43 ± 0.10 mg/dl (range being 0.20–0.70 mg/dl) and that for complicated appendicitis was 0.72 ± 0.13 mg/dl (range being 0.38–1.10 mg/dl), which was statistically significant (*p* < 0.05) rise of direct bilirubin with occurrence of complicated appendicitis. The mean Indirect Bilirubin for subjects with uncomplicated appendicitis was 0.36 ± 0.08 mg/dl (range being 0.16–0.60 mg/dl) and that for complicated appendicitis was 0.66 ± 0.14 mg/dl (range being 0.30–1.10 mg/dl), which was statistically significant (*p* < 0.05) rise of direct bilirubin with occurrence of complicated appendicitis.

There was significant rise in both components of total bilirubin, direct as well as indirect bilirubin in subjects with complicated appendicitis, so it was Mixed Type of Hyperbilirubinemia in gangrenous/perforated appendicitis. The sensitivity of Total serum bilirubin in predicting complicated appendicitis was found 91.43% (76.942% to 98.196%), where as the specificity of this test was 88.00% (78.439% to 94.363%). positive predictive value and negative predictive value were 78.03% and 95.65% respectively. Positive likelihood ratio and negative likelihood ratio were found to be 7.619 and 0.097 respectively taking prevalence of Complicated appendicitis be 31.80% (Table 2).

After plotting the True Positive Rate (TPR)/sensitivity of Total Serum Bilirubin level against the False Positive Rate (FPR) of its detection of complicated appendicitis at various threshold settings a ROC (Receiver Operating Characteristic) curve was obtained which shows optimal criterion at Total Bilirubin Level 1.06 mg/dl where sensitivity was 91.43% and specificity was 97.33% at 95% confidence interval with 31.8% disease prevalence (Table 2).

**Fig. 2:**
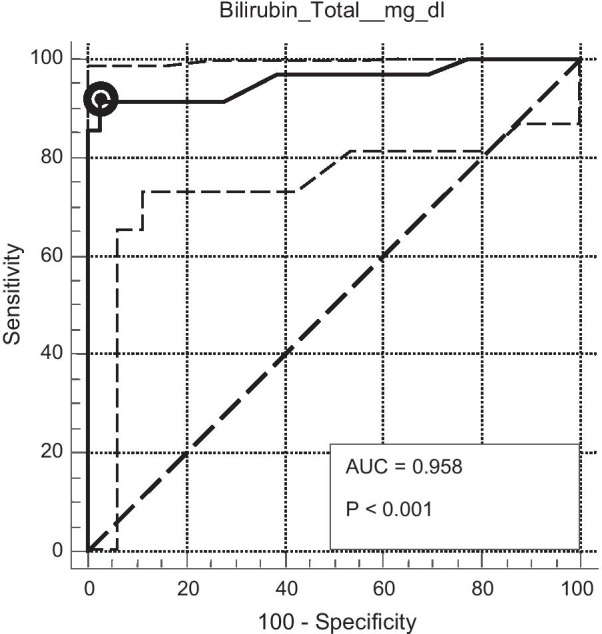
statistical significance of hyperbilirubinemia in complicated appendicitis with criterion values and coordinates of the roc curve (n = 110)

The prevalence of complicated appendicitis was found to be 31.8% among all cases. So Area under the ROC curve (AUC) was 0.958 [standard error 0.0246, 95% Confidence interval0.902 to 0.987, z statistic 18.614, Significance level *p* (area = 0.5) < 0.0001]. Figure 2 shows criterion values and coordinates of the ROC curve. This ROC (Receiver operating characteristic) curve reveals diagnostic ability of Total Serum Biliruin level for complicated appendicitis, after taking into consideration of prevalence of complicated appendicitis among total cases. Optimal criterion was found at total bilirubin level 1.06 mg/dl.

So we can infer that, subjects with clinical features suggestive of appendicitis with higher values of serum total bilirubin, are more susceptible of having complicated appendicitis than those with normal or mildly elevated total bilirubin level.

### Comparison of pre-operative and post-operative day 7 total bilirubin level

Serum total bilirubin level was checked in all study subjects irrespective of their final diagnosis on 7th Postoperative day to confirm that the hyperbilirubinemia was due to complicated appendicitis and it comes to normal range after appendectomy. It was seen that mean total bilirubin level was 1.39 mg/dl in complicated appendicitis group (n = 35) and 0.79 mg/dl in uncomplicated appendicitis group. Both the group showed mean serum total bilirubin value 0.69 mg/dl on 7th postoperative day. The preoperative mean total bilirubin of all the study subjects was 0.9841 mg which came to a mean value of 0.6885 mg/dl which was statistically significant (*p* < 0.05) (Table 2).

3 Relationship of other patient related parameters with complicated appendicitis

1. Alvarado score

The mean value for ALVARADO SCORE in Acute uncomplicated Appendicitis was 7.25 ± 0.71 (range 6–9) whereas for complicated appendicitis it is 8.5 ± 0.77 U/l (range 7–10 *p* value > 0.05) which is statistically significant. Only 5.33% subjects without complicated appendicitis had Mantrels Score more than mean value for complicated appendicitis cases. So it was concluded that MANTRELS SCORE were significantly high in appendicular gangrenous change/perforation cases, pre-operatively. And only 23% of all subjects with perforations (n = 13) had SCORE below that mean value for complication at presentation. Score above mean value 7.66 had a sensitivity of 94.28% and specificity of 70.66% for complications (Table 3). Area Under Curve was 0.825 (range 0.741 to 0.891), Positive Likelihood Ratio = 3.214 Negative Likelihood Ratio = 0.081, Positive Predictive Value = 59.980% and Negative Predictive Value = 96.367% at 31.800% complicated appendicitis disease prevalence.

2. Duration of pain

**Table 3:** Distribution of study population according to alvarado score at admission (n = 110)

Complicated appendicitis	Alvarado score					
	n (%)	Mean	SD	t	df	*p* value
Present	35(31.8)	8.5	0.77	8.85	108	< 0.05
Absent	75(68.2)	7.25	0.71			
Lowest value	6.0000					
Highest value	10.0000					
Arithmetic mean	7.6636					
Standard deviation	0.9605					
Median	7.5000					

	Complicated			Uncomplicated		
Alvarado score > mean (7.66)	33			22		
Alvarado score < mean (7.66)	2			53		
Total	35			75		

**Fig. 3:**
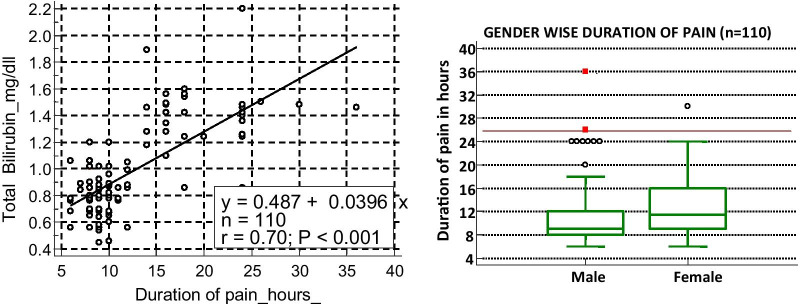
linear regression analysis of relationship between pain duration and total bilirubin and gender wise duration of pain (n = 110)

This study revealed relationship between mean duration of pain and complicated appendicitis among the study population. The mean duration of pain was shorter for subjects with an acute uncomplicated appendicitis (9.0 h ± 5.12 range 6–12 h) compared to those with a gangrenous/perforated appendix (20.2 ± 1.49 h, range 14–36 h), it reached statistical significance (*p* < 0.05).. The scattered plot diagram shows linear regression relationship between rise of total bilirubin level (0.0396 times) with increase of duration of pain (each hour) (Fig. 3).

### Gender wise distribution of pain duration

This study showed among 66 men mean duration of pain was 11.78 h ± 6.1 (range 6–36 h) while it is 13.75 h ± 5.9 (range 6–30 h) among 44 females (Fig. 3).

3. Temparature

40% of subjects with acute uncomplicated appendicitis and 71.42% of subjects with a gangrenous/perforated appendicitis presented with a fever. Statistical analysis revealed significant difference between two groups. And *p* value was < 0.05. The mean temperature in acute uncomplicated appendicitis was 98.02 + 1.30 °F (range 97–100 °F) whereas for complicated appendicitis it is 99 ± 1.49 °F (range 97–101 °F). Rise of temperature above mean value had sensitivity 71.43% (53.69% to 85.36%), specificity 60% (48.04% to 71.15%), Positive Likelihood Ratio of 1.78 and Negative Likelihood Ratio of 0.476 (Table 4).

4. Relationship between liver enzymes—alkaline phosphatage (ALP), aspartate amino transferase (AST), alanine amino transferase (ALT) and c reactive protein (CRP) level. with complicated appendicitis:

**Table 4:** Distribution of study population according to axillary temparature at admission (n = 110)

Complicated appendicitis	Temparature		Total	Χ^2^, df	*p* value
	< 98 °F	> 98 °F			
Absent	45	30	75	9.343, 1	< 0.05
Present	10	25	35		
Total	55	55	110		
Lowest value			97.0000		
Highest value			101.0000		
Arithmetic mean			98.3364		
95% CI for the arithmetic mean			98.0651 to 98.6076		
Median			98.0000		

The mean value for ALP in acute uncomplicated appendicitis was 126.04 ± 7.42 U/l (range 112–158 U/l) whereas for complicated appendicitis it was 125.85 ± 5.01 U/l (range 120–146 U/l *p* value > 0.05) which is not statistically significant. So it was concluded that ALP cannot differentiate between uncomplicated appendicitis and appendicular gangrenous change/perforation pre-operatively. And only 22.7% of all subjects (n = 110) had MILDLY elevated serum ALP level at presentation.

The mean value for AST in acute uncomplicated appendicitis was 33.70 ± 2.99 U/l (range 26–39 U/l) whereas for complicated appendicitis it was 32.88 ± 3.38 U/l (range 26–39 U/l *p* value > 0.05) which is not statistically significant. So it was concluded that AST cannot differentiate between uncomplicated appendicitis and appendicular gangrenous change/perforation pre-operatively. None (0%) of all subjects (n = 110) had elevated serum AST level at presentation.

The mean value for ALT in acute uncomplicated appendicitis was 33.34 ± 2.71 U/l (range 27–40 U/l) whereas for complicated appendicitis it was 32.77 ± 2.75 U/l (range 28–38 U/l *p* value > 0.05) which is not statistically significant. So it was concluded that ALT cannot differentiate between uncomplicated appendicitis and appendicular gangrenous change/perforation pre-operatively. And only 0.9% of all subjects (n = 110) had elevated serum ALT level at presentation.

So, this study showed isolated hyperbilirubinemia without much elevation in the liver enzymes. This isolated occurrence of hyperbilirubinemia was a significant predictor of gangrenous/perforated appendicitis.

90.7% of all uncomplicated Appendicitis had normal CRP level (< 0.6 mg/dl) whereas only 14% of complicated appendicitis subjects had normal CRP level. Rest 86% of complicated appendicitis subjects had statistically significant rise of CRP (> 0.6 mg/dl) which was raised only 9.3% of uncomplicated appendicitis cases. So it was concluded that raised CRP level can differentiate between uncomplicated appendicitis and appendicular gangrenous change/perforation pre-operatively (Table 5).

**Fig. 4:**
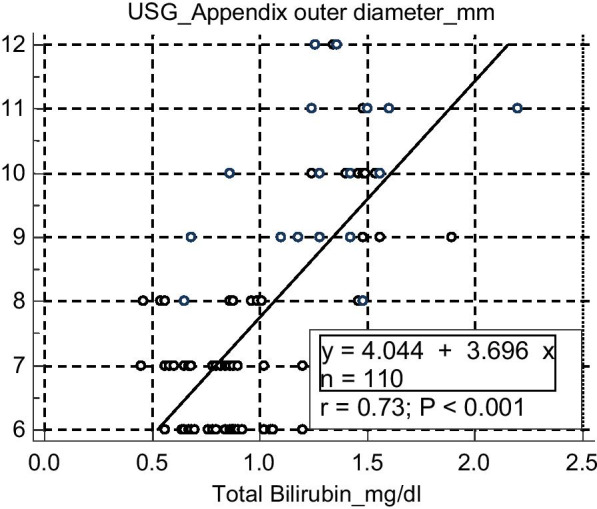
scattered plot diagram showing relationship between increase in appendix diameter with increase in serum total bilirubin level

5. Ultra sonographical findings of appendix:
  1. Appendix outer diameter on USG scan: The mean outer diameter of appendix of all study population was 7.6 mm. The mean appendix diameter on USG in uncomplicated Appendicitis was 6.64 mm ± 0.69 (range 6–8 mm) whereas for complicated appendicitis it was 9.71 mm ± 1.27 (range 7–12 mm, *p* value > 0.05) which was statistically significant. Sensitivity and specificity of increased appendix diameter on USG got sensitivity of 94.28% and specificity of 52.0%, Positive Likelihood Ratio 1.964, Negative Likelihood Ratio 0.110, Positive Predictive Value 47.805% and Negative Predictive Value 95.126% (Table 6). Scattered plot diagram showed one unit increase in appendix diameter was associated with 3.6% increase of total bilirubin level (Fig. 4). So it was concluded that increased appendix diameter on USG was associated with appendicular gangrenous change/perforation pre-operatively.
  2. 93.3% of all uncomplicated Appendicitis had clear appendix lumen on USG whereas only 11.4% of complicated appendicitis subjects had clear lumen. Rest 88.6% of complicated appendicitis subjects had appendix lumen obstruction by fecolith, evident on pre-operative USG. But only 6.7% of uncomplicated appendicitis cases had luminal obstruction. So it was concluded that luminal obstruction on USG can predict appendicular gangrenous change/perforation pre-operatively (Table 6).
  3. Statistically significant (*p* < 0.05) difference was found while assessing the cases, preoperatively on USG, for peri-appendix collection. No subjects with uncomplicated appendicitis (n = 75) had any peri-appendix fluid collection. But among subjects with complicated appendicitis, 77.1% had pri-appendix fluid collection and only 22.9% had no collection as was evident on USG scan.
6. Multivariate regression analysis of different parameters with occurrence of hyperbilirubinemia:

**Table 5:** Distribution of study population according to serum ALT, AST, ALP, CRP level at admission (n = 110)

Complicated appendicitis	ALT		Total	Χ^2^, df	*p* value
	< 40	> 40			
Absent	74	1	75	0.471,1	> 0.05
Present	35	0	35		
Total	109	1	110		

Complicated appendicitis	AST		Total	Χ^2^, df	*p* value
	< 40	> 40			
Absent	75	0	75	14.545,1	> 0.05
Present	35	0	35		
Total	110	0	110		

Complicated appendicitis	ALP		Total	Χ^2^, df	*p* value
	< 130 n(%)	> 130 n(%)			
Absent	57(76)	18(24)	75	0.217, 1	> 0.05
Present	28(80)	7(20)	35		
Total	85(77.3)	25(22.7)			

Complicated appendicitis	CRP		Total	*p* value	
	< 0.6 n(%)	> 0.6 n(%)			
Absent	68(90.7)	7(9.3)	75	*p* < 0.05	
Present	5(14)	30(86)	35	Χ^2^ = 61.802	
Total	73	37	110	Df = 1

**Table 6:** Distribution of study population according to mean appendix diameter, peri-appendix collection and presence of fecolith on USG (n = 110)

Complicated appendicitis	Mean appendix outer diameter on USG in two groups					
	n (%)	Mean diameter (mm)	SD	Χ^2^	df	*p* value
Abesent	75(68.2)	6.64	0.69	21.66	1	< 0.05
Present	35(31.8)	9.71	1.27			

	Distribution according to appendix diameter n(%)					
	< 7.6 mm	> 7.6 mm	Total	Χ^2^	df	*p* value
Abesent	33(94.29)	36(48)	69	21.66	1	< 0.05
Present	2(5.71)	39(52)	41			
Total	35(31.8)	75(68.2)	110			

	Peri-appendix collection					
	Present n(%)	Absent n(%)	Total	Χ^2^	df	*p* value
Absent	0(0)	75(100)	75	76.67	1	< 0.05
Present	27(77.1)	8(22.9)	35			
Total	27(24.5)	83(75.5)	110			

	Appendix lumen obstruction by fecolith n(%)					
	Present n(%)	Absent n(%)	Total	Χ^2^	df	*p* value
Absent	5(6.7)	70(93.3)	75	70.05	1	< 0.05
Present	31(88.6)	4(11.4)	35			
Total	36(32.72)	74(67.28)	110

Analysis of relationship of different patient related factors with occurrence of hyperbilirubinemia was done in this study (Table 7). According to the analysis non significant variables with occurrence of hyperbilirubinemia are—Age < 19 years = 1.236 (0.264–5.790), Gender-Female = 0.462 (0.124–1.714), duration of pain < 9 h = 0.955, Liver enzymes (viz. ALP, AST, ALT), C reactive Protein < 0.6 = 1.783, Appendix outer diameter < 8 mm = 1.620. The significant variable was complicated appendicitis = 0.01 (0.001–0.114).

**Table 7:** Multivariate regression analysis of different parameters with occurrence of complicated appendicitis

Parameters in relation to development of complicated appendicitis absent = 1, present = 2(1)	df	Significance (*p*)	Exp(B)	95% CI for Exp(B)	
				Lower	Upper
Duration of pain	1	0.957	0.955	0.177	5.157
CRP: < 0.6 = 1 > 0.6 = 2(1)	1	0.547	1.783	0.272	11.705
Appendix: < 7.6 mm = 1 > 7.6 mm = 2(1)	1	0.689	1.620	0.153	17.110
Hyperbilirubinemia	1	0.000	0.010	0.001	0.114
Constant	1	1.000	0.000		

## Discussion

Emergency appendectomy for uncomplicated acute appendicitis usually follows a short recovery period but same for the gangrenous or perforated appendicitis may be a life threatening one. To avoid morbidities prompt diagnosis is the key factor. Estimation of serum total bilirubin level is not commonly done as a marker of complicated appendicitis in an emergency setting. However, several previous studies have shown hyperbilirubinemia has high specificity for appendicular perforation. Comparison of sensitivity, specificity, PPV, NPV of serum total bilirubin in various studies including the present one has been depicted in Table 8. In adults, hyperbilirubinemia is commonly seen in liver or gallbladder diseases. Gilbert’s syndrome may also cause isolated indirect hyperbilirubinemia but its prevalence is 6% [38]. This prevalence is found considerably less than the incidence of Hyperbilirubinemia associated with acute appendicitis (evidenced both in previous studies as well as present study). The data was documented at the time of admission, so hyperbilirubinemia as a consequence of liver injury due to anesthetic agents, blood transfusion, or medication was excluded. Septic shock with subsequent ischemic injury to the hepatocytes has not occurred in any of them. The explanation for this hyperbilirubinemia associated with complicated appendicitis is circulating endotoxin related to the appendiceal infection. As explained in literature, in appendicitis, elevated intra-luminal pressure and ischemic necrosis of mucosa causes tissue gangrene or perforation. This is accompanied by bacterial cytotoxin facilitated progressive bacterial invasion. This elevated load of bacteria causes direct invasion or translocation into the portal system. Direct invasion of bacteria into the hepatic parenchyma interferes with the bilirubin excretion into the bile canaliculi biochemically rather than by any obstructive pathway [27].

**Table 8:** Comparison of sensitivity, specificity, ppv, npv of serum total bilirubin in various studies

Authors	Study type	Number of subjects	Age (years, mean, range)	Hystologically confirmed appendicitis (n)	Perforated appendix (n)	Sensitivity	Specificity	Positive likelihood ratio	Negative likelihood ratio
Present Study by Bakshi et al. 2019	Prospective non-randomized	110	23.5 years (5–62)	110	35	0.9143	0.88	7.619	0.097
Estrada et al. [7]	Retrospective non-randomized	170	33 years (5–66)	157	41	0.56	0.69	1.81	0.64
Emmanuel et al. [31]	Retrospective non-randomized	472	27 years (5–82)	386	45	0.60	0.70	1.99	0.57
Khan et al. [32]	Prospective non-randomized	122	29 years (8–73)	118	18	0.72	0.18	0.88	1.54
Sand et al. [33]	Retrospective non-randomized	538	36 years (6–91)	376	97	0.70	0.86	5.06	0.35
Käser et al. [34]	Retrospective non-randomized	1073	22 years (5–92)	725	155	0.38	0.78	1.71	0.8
Atahan et al. [35]	Retrospective non-randomized	351	31 years (18–83)	302	45	0.80	0.84	4.9	0.24
Hong et al. [36]	Retrospective non-randomized	977	31 years	732	245	0.32	0.84	2.01	0.81
McGowan et al. [37]	Retrospective non-randomized	1271	–	1053	154	0.55	0.90	5.76	0.50

More over in this present study pre-operative mean total bilirubin level in complicated appendicitis cases came down from 1.39 mg/dl (uncomplicated group had pre-operative mean of 0.79 mg/dl) to 0.69 mg/dl during post-operative evaluation on 7th post operative day, which was equal to those having uncomplicated appendicitis after similar duration. So it was clear that the hyperbilirubinemia occurred due to complicated appendicitis only. No previous studies had estimated this normal serum bilirubin level in postoperative follow up period (Table 2).

In search of relationship of different parameters with occurrence of complicated acute appendicitis, the present study revealed that greater duration of pain, higher Alvarado score, pyrexia are associated with more chances of gangrenous or perforated appendicitis. USG scan is also helpful in predicting complicated appendicitis. The mean outer appendix diameter was 9.71 mm in case of complicated appendicitis. Presence of fecolith and peri-appendix collection were also associated with increased chances of complicated appendicitis. Summary of all parameters from present study is depicted in Table 9.

**Table 9:** Summary of all parameters from present study

Parameter	Type of appendicitis	Mean	SD	*p* value
AGE (years)	Uncomplicated	26.81	13.22	< 0.05
	Complicated	16.48	14.90	
Total bilirubin (mg/dl)	Uncomplicated	0.79	0.16	< 0.05
	Complicated	1.39	0.26	
Direct bilirubin (mg/dl)	Uncomplicated	0.43	0.1	< 0.05
	Complicated	0.72	0.13	
Indirect bilirubin (mg/dl)	Uncomplicated	0.36	0.08	< 0.05
	Complicated	0.66	0.14	
ALP (mg/dl)	Uncomplicated	126.02	6.79	> 0.05
	Complicated	125.85	7.11	
AST (mg/dl)	Uncomplicated	33.7	3.05	> 0.05
	Complicated	32.88	3.38	
ALT (mg/dl)	Uncomplicated	33.34	2.76	> 0.05
	Complicated	32.77	2.52	
Alvarado score (1–10)	Uncomplicated	7.25	0.71	< 0.05
	Complicated	8.5	0.77	
Duration of pain (hours)	Uncomplicated	9.0	5.12	< 0.05
	Complicated	20.2	1.49	
Temparature (°F)	UNCOMPLICATED	98.02	1.42	< 0.05
	Complicated	99	1.47	
Appendix diameter (mm)	Uncomplicated	6.64	0.69	< 0.05
	Complicated	9.71	1.27	

So, hyperbilirubinemia is possible in complicated acute appendicitis.

There were some limitations of this study also, like-

1. Study population was limited to a specific geographical area. So to study the universal nature of the relationship between study variables, multi center trial should be done. Results of this study should be corroborated by larger studies.
2. Information regarding use of antibiotics before admission was not available in 3 (uncomplicated) cases.
3. Relationship was found between increase in total bilirubin level and complicated appendicitis in logistic regression analysis but supplementing them with other parameters along with the total bilirubin level will diagnose the complicated appendicitis more efficiently.

## Conclusion

This is to conclude that Serum Total Bilirubin level estimation, which is a simple, cheap and easily available laboratory test, can be added to the routine investigations in clinically suspected cases of acute appendicitis. The rise in serum bilirubin level in subjects with acute appendicitis should be considered as having higher probability of complication (gangrene or perforation). Together with clinical findings and other routine laboratory tests, presence of serum hyperbilirubinemia may help in managing subjects with complicated acute appendicitis earlier. The role of serum hyperbilirubinemia, as a new diagnostic marker of complicated appendicitis, may be of particular help in

1. In surgical resource poor areas (like in ships, mountains, remote areas) suspected appendicitis cases with elevated serum bilirubin level should seek early surgical help.
2. In atypical presentation of acute appendicitis (like in retrocecal appendicitis, retroperitoneal appendicitis) hyperbilirubinemia should raise suspicion of complications.

Further studies may be carried out to assess the usefulness of hyperbilirubinemia as a diagnostic marker in acute complicated appendicitis with certain situations, like

1. In case of advanced pregnancy (atypical location of appendix).
2. In case of doubtful condition regarding appendicular lump formation and lack of confirmatory radiological facility, presence of serum hyperbilirubinemia may point towards complicated appendicitis and should necessitate surgical intervention.
3. In immunosuppressive condition (like transplant subjects, subjects on chemotherapy, AIDS, immunosuppressive drugs users), diabetic subjects with masked presentation of acute appendicitis, presence of hyperbilirubinemia may nessecitate surgical intervention.

## Data Availability

The datasets used and/or analysed during the current study are available from the corresponding author on reasonable request.

## Abbreviations

USG: Ultra-sonography
MDCT: Multi-detector computed tomography
CRP: C reactive protein
OPD: Out patient department
PPV: Positive predictive value
NPV: Negative predictive value
